# Advances in Computer Vision and Spectroscopy Techniques for Non-Destructive Quality Assessment of *Citrus* Fruits: A Comprehensive Review

**DOI:** 10.3390/foods14030386

**Published:** 2025-01-24

**Authors:** Kai Yu, Mingming Zhong, Wenjing Zhu, Arif Rashid, Rongwei Han, Muhammad Safiullah Virk, Kaiwen Duan, Yongjun Zhao, Xiaofeng Ren

**Affiliations:** 1 School of Food and Biological Engineering, Jiangsu University, Zhenjiang 212013, China; yukai317@163.com (K.Y.); mingming.zhong@ujs.edu.cn (M.Z.); arif_ktk007@hotmail.com (A.R.); safiullahvirk@hotmail.com (M.S.V.); z719503413@163.com (Y.Z.); 2 School of Agricultural Engineering, Jiangsu University, Zhenjiang 212013, China; 2222216044@stmail.usj.edu.cn; 3 College of Food Science and Engineering, Qingdao Agricultural University, Qingdao 266109, China; qauhan@qau.edu.cn; 4 Institute of Food Physical Processing, Jiangsu University, Zhenjiang 212013, China

**Keywords:** *Citrus* fruit, computer vision, spectroscopy, data fusion, non-destructive quality assessment

## Abstract

*Citrus* fruits, classified under the Rutaceae family and *Citrus* genus, are valued for their high nutritional content, attributed to their rich array of natural bioactive compounds. To ensure both quality and nutritional value, precise non-destructive testing methods are crucial. Among these, computer vision and spectroscopy technologies have emerged as key tools. This review examines the principles and applications of computer vision technologies—including traditional computer vision, hyperspectral, and multispectral imaging—as well as various spectroscopy techniques, such as infrared, Raman, fluorescence, terahertz, and nuclear magnetic resonance spectroscopy. Additionally, data fusion methods that integrate these technologies are discussed. The review explores innovative uses of these approaches in *Citrus* quality inspection and grading, damage detection, adulteration identification, and traceability assessment. Each technology offers distinct characteristics and advantages tailored to the specific testing requirements in *Citrus* production. Through data fusion, these technologies can be synergistically combined, enhancing the accuracy and depth of *Citrus* quality assessments. Future advancements in this field will likely focus on optimizing data fusion algorithms, selecting effective preprocessing and feature extraction techniques, and developing portable, on-site detection devices. These innovations will drive the *Citrus* industry toward increased intelligence and precision in quality control.

## 1. Introduction

*Citrus* refers to a group of fruits within the genus *Citrus*, part of the Rutaceae family, including species such as mandarin orange, orange, grapefruit, lemon, lime, and citron [1,2,3]. These plants originated in the tropical and subtropical regions of Asia and Oceania and are now among the most widely cultivated and popular fruit crops globally, with *Citrus* planting area and production volume leading the world. China holds the top position in overall *Citrus* production, while the United States and Brazil are the largest producers of oranges [4,5]. *Citrus* fruits are nutrient-rich, containing carbohydrates, organic acids, vitamins, minerals, and dietary fiber, as well as natural bioactive compounds like pectin, flavonoids, and carotenoids [5,6]. These nutrients are vital for maintaining human health, and their appealing flavor and substantial health benefits make them highly popular among consumers [7].

As market demand for *Citrus* grows, quality characteristics influencing consumer choices have become increasingly important. *Citrus* quality assessment at harvest generally considers two dimensions: external attributes (e.g., color, size, shape, and visible defects) and internal qualities (e.g., soluble solids content, acidity, maturity, and firmness) [8]. Traditional assessments rely on manual inspection and chemical testing, which are often destructive, time-consuming, costly, and subjective, limiting their ability to support a consistent fresh *Citrus* supply [9,10]. With labor shortage in agriculture, the *Citrus* industry requires fast, non-destructive, and cost-effective technologies. Non-destructive testing (NDT) methods, such as computer vision and spectroscopy, have shown promise due to their accuracy, cost-efficiency, and minimal sample preparation needs [11,12]. However, current NDT methods are generally limited in scope. Integrating computer vision with spectroscopy offers a pathway to multivariate, high-precision *Citrus* quality assessments, advancing the industry toward more comprehensive and efficient quality control.

The application of computer vision and spectroscopy in *Citrus* quality assessment and grading has been partially addressed in prior reviews. For instance, Peng et al. examined the use of machine vision for detecting *Citrus* pests and diseases, as well as for harvest identification and grading [13]. Palei et al. reviewed the current research landscape, limitations, and recommendations in *Citrus* disease detection and fruit grading [14]. Dhiman et al. assessed various classification models for visual disease detection in *Citrus* fruits [15]. Additionally, Cavaco et al. explored the use of Vis/NIR spectroscopy in assessing *Citrus* fruit quality and maturity, broadening the research scope in this field [8]. However, these reviews either focus solely on computer vision or only consider spectroscopy applications for internal quality detection, lacking a comprehensive exploration of their combined use.

This paper addresses this gap by reviewing recent advancements in both computer vision and various spectroscopic techniques for non-destructive *Citrus* quality assessment. It provides an overview of the basic principles, typical configurations, and primary applications of these technologies. Furthermore, the paper briefly introduces the data-processing processes in computer vision and spectroscopy, and it discusses the applications of these non-destructive technologies in *Citrus* quality detection and grading, damage detection, *Citrus* adulteration identification, and ensuring traceability. Finally, this paper analyzes the characteristics of computer vision and spectroscopy technologies in *Citrus* quality evaluation, discusses the advantages and disadvantages of using different data fusion techniques in *Citrus* quality detection, and explores the future directions and prospects of *Citrus* quality assessment.

## 2. Computer Vision Techniques

Computer vision (CV) is a technology that enables computers to analyze and process visual information, drawing on principles from image processing, signal processing, neural networks, and machine learning [16]. This field integrates multiple disciplines, including image processing, computer science, and pattern recognition, exemplifying the nature of interdisciplinary research. With technological advancements, CV has expanded to encompass traditional computer vision, as well as multispectral and hyperspectral imaging techniques.

### 2.1. Traditional Computer Vision Techniques

**Figure 1:**
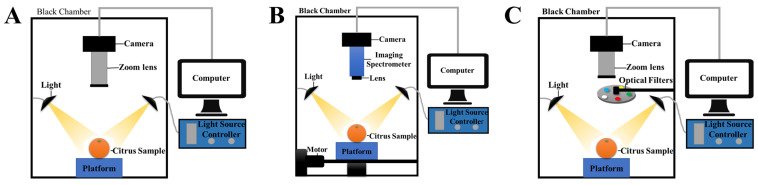
Simplified scheme of a computer vision system: (**A**) traditional computer vision, (**B**) hyperspectral imaging, and (**C**) multispectral imaging.

Traditional CV technology is a rapidly developing branch of artificial intelligence that utilizes computers and cameras to replace conventional visual measurement and assessment methods [13]. A classical CV system consists of illumination devices, image acquisition equipment (including cameras and lenses), image acquisition cards, and computers, which respectively provide functions of uniform illumination, image acquisition, and image processing [17]. The simplified scheme of the CV system is shown in Figure 1A. This technology converts the acquired target data into image signals using Charge-Coupled Device (CCD) or Complementary Metal–Oxide–Semiconductor (CMOS) cameras and transmits them to computers to obtain digital morphological information of the objects to be measured, thereby enabling measurement, classification, and recognition of the target data [18,19].

CV technology has been widely adopted in the external inspection of *Citrus* fruits due to its simplicity, low cost, and high detection efficiency. This technology not only precisely detects the external quality characteristics of *Citrus* fruits, such as color, size, shape, and texture, enabling efficient grading and classification [20]; it also facilitates the measurement of *Citrus* weight [21] and volume [22] through visual methods. Furthermore, changes in *Citrus* peel color can be utilized to assess the maturity of the fruit [23]. In practical applications, CV technology accurately identifies *Citrus* fruits within complex tree canopies, thereby providing a robust foundation for fruit-picking systems [24]. Additionally, this technology detects physical defects in *Citrus* fruits and classifies them [25]. In detecting *Citrus* diseases, such as Huanglongbing (HLB) [26], Anthracnose [27], Canker [28], and Black Spot [29], and performing later-stage detection of *Citrus* decay [30], this technology has demonstrated remarkable accuracy, making significant contributions to reducing losses and ensuring steady market supply.

### 2.2. Hyperspectral Imaging Technology

Hyperspectral imaging (HSI) technology combines spectroscopy, imaging science, and image-processing techniques, merging two-dimensional imaging with spectral technologies [31,32]. It captures spectral data across multiple contiguous frequency bands in the 200–2500 nm range for each pixel, thus providing rich spectral features [33,34]. HSI systems integrate both imaging and spectroscopy into a single platform, enabling the simultaneous acquisition of spatial image and spectral data. A typical HSI setup includes a light source, sample platform, imaging spectrometer, camera, computer with data-processing software, and motion control devices, all contained within a black chamber [35], as shown in Figure 1B. Various light sources, such as halogen lamps, LEDs, and lasers, are used in HSI systems [36]. Light is dispersed by a series of wavelength-dispersion devices before being captured by the camera, and the resulting data are processed on the computer [37].

In the *Citrus* industry, HSI technology has become essential for detecting both the quality and the internal and external defects, facilitating quick identification of damage [37,38]. It detects external characteristics by acquiring spatial data on shape, size, and visible defects while simultaneously measuring internal quality attributes through spectral reflectance or transmittance across various wavelength bands [39]. These internal features include soluble solids content (SSC) [35], sugar content [40], acidity [40], and flavonoid compound [41], which are critical for assessing and grading *Citrus* quality. Additionally, analyzing the distribution of nutritional components helps determine quality grade and ripeness [42]. HSI technology also allows for the rapid detection of both internal and external defects, facilitating quick identification of damage [38]. When integrated with Geographic Information Systems (GISs) and Global Positioning Systems (GPSs), HSI can be used to monitor conditions like chlorophyll deficiency, water status [43], and disease prevalence [44] in *Citrus* orchards, supporting plant health monitoring, yield enhancement, and disease prevention and control [45]. In conclusion, HSI technology is vital in the *Citrus* industry for non-destructive quality assessment, detecting both internal and external defects, and enhancing grading, making it an essential tool for optimizing *Citrus* production.

### 2.3. Multispectral Imaging Technology

Multispectral imaging (MSI) is a spectral imaging technique that captures reflection or emission spectra in a few discrete bands, such as visible light and near-infrared, to create images [46,47]. The hardware configuration of MSI systems is similar to that of hyperspectral systems. Still, MSI typically uses 3 to 20 spectral bands, resulting in lower requirements for optical design, sensor accuracy, and data-processing capabilities. In addition to commercial multispectral cameras, spectral filters are commonly employed for band selection, as shown in Figure 1C [48].

The reduced number of bands and simpler data processing make MSI systems more efficient and cost-effective, especially for large-scale remote-sensing applications [49]. MSI is well-suited for tasks that require spectral information without excessive detail, such as monitoring *Citrus* vegetation [50], detecting water stress [51], identifying *Citrus* defects [4], assessing *Citrus* maturity [52], and detecting diseases [53].

### 2.4. Summary of Computer Vision and Related Imaging Technologies

Other imaging techniques, including micro X-ray fluorescence imaging (micro-XRF), Raman imaging, fluorescence imaging, magnetic resonance imaging (MRI), X-ray imaging, and thermal imaging, have also been explored for *Citrus* quality assessment. Specifically, Tian et al. used micro-XRF imaging technology to observe the impact of HLB disease on the distribution of elements such as Zn in *Citrus* plant leaves, finding a significant reduction in Zn concentration in leaves infected with HLB [54]. Cai’s team successfully characterized and identified fungal decay in *Citrus* fruits using Raman scattering spectral imaging [55]. Siregar et al. employed fluorescence imaging to detect mechanical damage in *Citrus* fruits [56]. Zur et al. utilized MRI to predict fruit splitting in Nova *Citrus*, achieving predictions up to two months prior to the actual occurrence of splitting [57]. Hsiao’s team constructed visual grayscale images using X-ray imaging technology and analyzed changes in lemon quality and maturity through quantitative statistical methods [58]. Additionally, Gan and his team developed an active thermal imaging system that accurately estimates the number of unripe fruits based on thermal images of *Citrus* tree canopies [59]. These research findings not only enrich the application of computer vision technology in the agricultural field but also provide powerful technical support for quality inspection and disease prevention in *Citrus* and other fruits.

## 3. Spectroscopy Techniques

Spectroscopy techniques are crucial in NDT for *Citrus* quality evaluation. These methods are non-invasive, fast, and highly accurate, offering precise insights into the chemical composition of *Citrus* fruits, including SSC, titratable acidity (TA), and vitamins [60]. Common spectroscopic techniques include infrared spectroscopy, Raman spectroscopy, fluorescence spectroscopy, terahertz spectroscopy, and nuclear magnetic resonance spectroscopy. Each of these techniques possesses unique detection principles and applicable ranges, collectively providing robust technical support for the precise evaluation of fruit quality.

### 3.1. Infrared Spectroscopy

**Figure 2:**
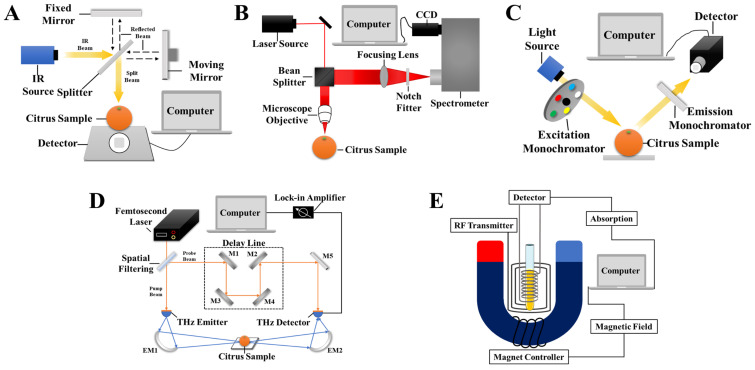
Simplified scheme of different spectral systems: (**A**) infrared spectroscopy, (**B**) Raman spectroscopy, (**C**) fluorescence spectroscopy, (**D**) terahertz spectroscopy (M1–M5: reflecting mirrors, EM1–EM2: off-axis elliptic mirrors), and (**E**) nuclear magnetic resonance spectroscopy.

Infrared spectroscopy (IR) is a spectral analysis technique based on the principle of molecular vibrational interactions. Different organic molecules in *Citrus* samples possess unique vibrational modes. When these molecules are excited by infrared radiation, they absorb or scatter light at specific wavenumbers or wavelengths, producing characteristic spectra [61]. IR spectroscopy is divided into three distinct regions according to the electromagnetic spectrum: the near-infrared (NIR) region, spanning from 780 nm to 2500 nm; the mid-infrared (MIR) region, ranging from 2500 nm to 25,000 nm; and the far-infrared (FIR) region, extending from 25,000 nm to 1,000,000 nm. An IR system typically consists of a light source, a spectral dispersing device, a sample chamber, a detector, and a computer data-processing system [62], as shown in Figure 2A. Halogen lamps effectively excite the sample and generate spectral information. The spectral dispersing device decomposes the infrared radiation emitted by the source into components of different wavelengths, which then interact with the sample in the sample chamber. Common detectors convert optical signals into electrical signals, which are transmitted to the computer data-processing system for analysis and processing, ultimately producing the infrared spectral data of the sample.

IR spectroscopy is widely used to analyze various components in *Citrus* fruits, such as SSC [63], acidity [11], vitamin content [64], and flavonoids [65], providing essential data for the quality grading and classification of *Citrus*. Additionally, changes in infrared spectral absorption and scattering can help detect defects [66] and identify biochemical changes caused by diseases. This capability offers crucial technical support for early disease detection, enabling timely intervention to reduce losses and improve disease management strategies [67]. This information plays a crucial role in assessing fruit quality, optimizing cultivation management, and guiding market sales.

Visible–near infrared (Vis-NIR) spectroscopy, which covers the range from 400 nm to 2500 nm, combines both visible and near-infrared bands, and it captures color information in the visible spectrum while revealing internal chemical and physical properties of materials via the spectral characteristics of the NIR band [8,68]. Vis-NIR spectroscopy is valuable for detecting parameters such as sugar content, acidity, SSC, maturity, and defects in *Citrus* fruits [69].

### 3.2. Raman Spectroscopy

Raman spectroscopy is based on the principle of Raman scattering [70]. A laser source is directed at sample molecules, causing the laser light to scatter off the molecular bonds of the analyte. The resulting inelastic scattered light is then collected and processed to generate the Raman spectrum [71]. A Raman spectroscopy system primarily consists of a laser source, a sample chamber, optics, a spectral dispersion device, a detector, and a computer data-processing system [72]. Commonly used laser sources include helium–neon lasers (He-Ne) and diode lasers, which excite the sample and generate Raman scattering. The spectral dispersion device separates the scattered light into different wavelengths, with common devices including diffraction gratings and interferometers. The detector converts the scattered light into electrical signals, which are processed and visualized by the computer system to yield the Raman spectral data. A simplified scheme of the Raman spectroscopy system is shown in Figure 2B.

Raman spectroscopy has demonstrated significant advantages in analyzing chemical components in *Citrus* fruits, enabling quantitative analysis of sugars, acidity, carotenoids, and flavonoids, among other antioxidant substances [73,74]. This provides a scientific basis for assessing the maturity and freshness of *Citrus* fruits [75], as well as for variety classification [76]. Surface-enhanced Raman spectroscopy (SERS) technology provides a more precise means for the detection of trace components in *Citrus* fruits, including the detection of pesticide residues in *Citrus* [72,77,78]. The combination of micro-imaging technology with SERS allows for a deeper understanding of changes in the chemical structure within fruit tissues [73]. Additionally, Raman spectroscopy can be utilized to detect diseases in *Citrus*, such as fungal infections [55] or HLB [79]. In summary, Raman spectroscopy is a powerful technique for analyzing chemical components and detecting diseases in *Citrus* fruits.

### 3.3. Fluorescence Spectroscopy

Fluorescence is the emission of light from fluorophore after the absorption of UV or VIS light [80]. The principle of fluorescence spectroscopy involves the absorption of light by atoms or particles within a substance (e.g., ultraviolet light), causing them to transition to an excited state and subsequently releasing energy in the form of light at a longer wavelength [81,82]. A typical fluorescence spectroscopy system consists of a light source, sample chamber, optical system, detector, and data-processing system, as shown in Figure 2C. Light sources such as LEDs, xenon lamps, and lasers emit stable, specific wavelengths of light to excite the sample. The sample chamber holds the sample, while the optical system includes lenses, filters, spectrometers, and other components that focus and disperse both excitation and fluorescence light, ensuring signal clarity. Detectors like photomultiplier tubes (PMTs), filter photodiodes, and CCDs capture the fluorescence signals. The data-processing system analyzes and processes the data to measure fluorescence intensity and wavelength, facilitating the generation of charts and reports for accurate interpretation.

Fluorescence spectroscopy, as a spectral technique, holds significant potential in *Citrus* fruit quality assessment and detection due to its high sensitivity and non-destructive nature. *Citrus* fruits contain various fluorescent compounds, including chlorophyll, flavonoids, and carotenoids, which contribute to their characteristic fluorescence [81]. Fluorescence spectroscopy is highly effective in measuring the fluorescence intensity of these compounds, which can be used to assess key parameters, such as SSC, acidity [83], and vitamin C content, in *Citrus* fruits. At different maturity stages, *Citrus* fruits exhibit distinct fluorescence characteristics, allowing for the inference of their nutritional composition and ripeness based on fluorescence signals [83,84,85]. In summary, fluorescence spectroscopy demonstrates significant importance and broad application potential in various fields, including *Citrus* quality assessment.

### 3.4. Terahertz Spectroscopy

Terahertz (THz) spectroscopy operates within the frequency range from 0.1 THz to 10 THz [61]. It is a novel detection technology that combines the properties of both microwaves and infrared radiation, offering low photon energy and strong penetration capabilities [86,87]. These unique characteristics make THz spectroscopy particularly well-suited for identifying intermolecular interactions and detecting subtle vibrational modes within both intermolecular and intramolecular structures, thereby providing rich vibrational information [88]. A typical THz spectroscopy system consists of a light source, a terahertz radiation emitter, a sample chamber, a detector, optical components, a computer, and spectral processing software. Figure 2D illustrates the configuration of a THz spectroscopy system. The light source often includes photoconductive antennas, quantum cascade lasers (QCLs), or THz pulse sources, which emit THz radiation. The radiation is directed onto the sample through optical components. The interaction of the THz radiation with the sample can result in absorption, scattering, or transmission, which is then detected by sensors, like superconducting bolometers or photoconductive detectors. These detectors convert the detected signals into electrical signals, which are subsequently processed by a computer.

Due to its exceptional penetration ability, THz spectroscopy allows for the non-invasive analysis of the internal structure and composition of *Citrus* fruits, including the detection of sugars, flavonoids [88], and vitamin C content. This capability provides valuable support for assessing the overall quality of *Citrus* fruits. Additionally, THz spectroscopy is sensitive to water content, making it useful for measuring moisture levels, detecting freeze injury, and assessing low-temperature stress in *Citrus* fruits [89]. It also has been applied to detect biological and chemical substances, such as carbendazim in oranges [87]. Therefore, it opens up new possibilities for quality control and advanced scientific management in the *Citrus* industry.

### 3.5. Nuclear Magnetic Resonance Spectroscopy

Nuclear magnetic resonance (NMR) spectroscopy is based on the interaction of atomic nuclei with electromagnetic radiation when exposed to a uniform external magnetic field [49]. An NMR system typically consists of a magnet system, radiofrequency transmission and reception components, a sample chamber, a detection coil, a computer system, and data-processing software, as shown in Figure 2E. These components work together to enable nuclei with magnetic moments to absorb RF energy at specific frequencies under a magnetic field, undergoing energy transitions and emitting NMR signals. These signals are captured and processed to provide detailed information about the type, quantity, and chemical environment of the nuclei within the *Citrus* fruits sample.

NMR technology is particularly effective in detecting a wide range of components in *Citrus* fruits, such as sugars, amino acids, organic acids, and flavonoids [90,91,92]. It enables precise classification based on variety, geographical origin [93], and even the presence of adulteration [94]. This capability is essential for evaluating the quality, maturity [95], and chemical composition of *Citrus* varieties [92]. Moreover, NMR plays a crucial role in detecting and preventing *Citrus* diseases [96]. It not only accurately diagnoses *Citrus* diseases, but when combined with metabolomics analysis, it also identifies metabolic changes associated with these diseases [97]. Additionally, NMR helps monitor changes in key chemical components during *Citrus* growth, providing insights into plant health, growth status, and internal dynamics, which are valuable for variety optimization and improving cultivation practices [95]. In summary, NMR spectroscopy technology exhibits significant application value in various aspects, including *Citrus* quality assessment, disease detection, and cultivation management.

### 3.6. Summary of Spectral Technologies

Several other spectral-based techniques can also be used for *Citrus* detection, including time-resolved spectroscopy [98] and Laser-Induced Breakdown Spectroscopy (LIBS) [99]. Kurata et al. predicted the SSC and acidity of grapefruits by analyzing the changes in time-resolved curves and compared the results with those obtained using traditional NIR methods [98]. The findings indicated that the prediction accuracy was higher than that of conventional NIR measurements. Yao et al. achieved 100% detection accuracy for *Citrus* HLB by combining LIBS technology with Principal Component Analysis (PCA) and a Multilayer Perceptron Neural Network model [99]. With its high efficiency, accuracy and non-destructive detection characteristics, spectroscopy technology plays an irreplaceable role in the internal quality inspection of the *Citrus* industry, providing strong technical support for improving quality, ensuring *Citrus* fruits’ safety, and promoting the healthy development of the industry.

## 4. Computer Vision Analysis and Chemometrics

### 4.1. Computer Vision Analysis

Computer vision techniques are commonly utilized for the detection and classification of *Citrus* fruits, involving steps such as image preprocessing, feature extraction, and classifier training. Image preprocessing encompasses denoising, image enhancement, color transformation, and segmentation, with the objective of enhancing the accuracy of subsequent processing stages [100]. In the feature extraction phase, methodologies such as the Histogram of Oriented Gradients (HOG), Local Binary Patterns (LBPs), and color histograms are utilized to extract *Citrus*-related features, including color, size, shape, texture, and defects. Simultaneously, these methods facilitate the separation of the target from the background, thereby aiding the classification process [101]. Regarding image modeling, traditional machine-learning approaches, such as support vector machine (SVM), Random Forest (RF), K-Nearest Neighbor (KNN), and Decision Tree (DT), rely heavily on manually extracted features. Consequently, they are well-suited for classification tasks that involve low-dimensional or straightforward features. However, these methods often struggle to capture intricate patterns and nonlinear characteristics [102]. In stark contrast, deep-learning models, notably Convolutional Neural Network (CNN) and ResNet, automatically extract complex features from raw images. These models exhibit exceptional performance in managing diverse datasets and achieving high-precision classification. Furthermore, object detection models, such as YOLO, possess the capability to swiftly locate and classify *Citrus* fruits.

### 4.2. Chemometrics

Chemometrics utilizes mathematical and statistical methods to analyze spectral data and extract information related to target properties, primarily consisting of three components: data preprocessing, feature extraction, and modeling. Spectral data preprocessing minimizes interference, enhances data consistency, and highlights relevant information, thus laying the foundation for subsequent modeling. Common preprocessing methods include baseline correction, Savitzky–Golay filtering (SG), normalization, Standard Normal Variate transformation (SNV), and Multiplicative Scatter Correction (MSC), as well as first- and second-order derivative methods. The choice of preprocessing techniques depends on the characteristics of the data, and studies have shown that combining multiple preprocessing methods can further enhance data quality. High-dimensional spectral data typically contain significant redundant information, and dimensionality reduction and feature selection are key to improving modeling efficiency and accuracy. Common dimensionality reduction techniques include Principal Component Analysis (PCA) and Partial Least Squares (PLS), while discriminant analysis methods, such as Linear Discriminant Analysis (LDA), and feature selection algorithms, like Successive Projections Algorithm (SPA) and Genetic Algorithm (GA), are also employed. Deep-learning techniques, such as CNN, have shown potential in feature extraction [49]. The most suitable technique should be selected based on the specific features of the *Citrus* fruit. Chemometric modeling establishes quantitative relationships between spectral data and target chemical properties (such as SSC, TA, and moisture content) and performs excellently in *Citrus* quality grading and defect detection. Regression methods, such as Partial Least Squares Regression (PLSR), Principal Component Regression (PCR), and Support Vector Regression (SVR), are commonly used for quantitative prediction, while classification methods, including Discriminant Analysis (DA), SVM, and KNN, are employed for quality grading. Multivariate calibration models based on chemometrics, such as Partial Least Squares Discriminant Analysis (PLS-DA) and multi-class SVM, effectively analyze the internal and external quality of *Citrus* fruits and have been successfully applied to predict SSC, TA, and damage defects.

## 5. Quality Detection Applications for *Citrus* Fruits

### 5.1. Citrus Quality Detection and Grading

Both external and internal characteristics determine the quality of *Citrus* fruits. External quality includes factors such as color, size, weight, shape, external damage, and the presence of diseases. In contrast, internal quality encompasses parameters like SSC, TA, ripeness index, firmness, and internal damage. The classification and grading of *Citrus* fruits can be based on these external and internal attributes, either individually or through a combination of both [103].

#### 5.1.1. *Citrus* External Quality Detection

The external appearance of *Citrus* fruits, as the first impression for consumers, directly influences marketability [104]. As a result, external quality is a critical factor in determining the desirability of *Citrus* fruits [64]. The primary external characteristics influencing consumer purchasing decisions include color, size, shape, texture, and defects [8].

Currently, the visual features of *Citrus* fruits, such as color, shape, and texture, can be represented by digital color images represented in the form of a matrix of RGB channel values in CV technology. By extracting the relevant features from these images, multivariate models can be applied for classification, recognition, and prediction of *Citrus* fruit quality. For example, S. Benallie et al., employed CV techniques to evaluate the color, size, and firmness of bergamot peel, achieving a classification accuracy of 78.86% using LDA, demonstrating the efficacy of computer vision for classifying external features of *Citrus* fruits [20]. The variation in peel color of *Citrus* fruits not only reflects varietal characteristics but also correlates with fruit maturity. During ripening, changes in internal components impact peel color, which is closely associated with flavor. Thus, peel-color changes can be used to assess both fruit maturity and flavor. For instance, Barkah’s study successfully utilized peel color to determine the maturity and flavor of Pontianak Siam oranges [23]. Furthermore, the relationship between peel color and sweetness—critical indicators of fruit quality—has been explored. Al-Sammarraie et al. studied the correlation between the RGB values of oranges and their sweetness, identifying the machine-learning algorithm with the highest predictive accuracy [105]. This underscores the potential of artificial intelligence in fruit quality assessment, offering valuable support for quality control in related industries and enhancing consumer satisfaction.

Accurate measurement of fruit size is essential in *Citrus* quality assessment, typically involving the evaluation of dimensions, volume, and weight. Non-contact measurement methods have been developed to facilitate this process. For example, Wang et al. employed the YOLOv5 model for rapid and accurate fruit recognition, achieving an accuracy rate of 95.6% [106]. To overcome occlusion issues caused by branches and leaves, they also implemented Cycle GAN technology, achieving an overall error of 10.12%, which meets high-throughput detection requirements. In comparison to 2D vision techniques, 3D reconstruction offers superior accuracy for determining fruit volume. Jadhav et al. utilized 3D reconstruction in a multi-camera setup to classify *Citrus* fruits based on volume and maturity characteristics, ensuring reliable results [22]. Weight measurement is another critical aspect of *Citrus* fruit grading. Phate et al., developed a CV system to correlate weight with physical attributes, employing models such as Dimension Analysis (DA), Normal Regression (NR), and Feedforward Artificial Neural Networks (FFANNs) to predict fruit weight. The NR model exhibited the best performance [21]. In subsequent research, they introduced a weight estimation model using a polynomial kernel SVM classifier and an optimized Adaptive Neuro-Fuzzy Inference System (ANFIS), providing robust support for the design of sorting, grading, and packaging systems for sweet oranges [107].

In automated fruit classification, visual systems are often integrated with conveyor belts, forming advanced sorting systems that classify and grade fruits based on color, size, shape, and texture [108]. This significantly reduces labor costs [9]. Chakraborty et al. modified the lightweight Deep Convolutional Neural Network (DCNN) model “SortNet”. They deployed it on edge devices, achieving real-time classification and weight grading of *Citrus* fruits with accuracies of 97.0% and 91.3%, respectively. This system provides strong support for automation in packaging operations [9]. In the design of grading systems, it is crucial to balance performance, cost, power consumption, and hardware capabilities. M.A. Núño-Maganda et al., proposed a visual system based on a Field-Programmable Gate Array (FPGA) hardware architecture. They used DT algorithms for classification, demonstrating excellent performance [109]. By combining multiple features, the accuracy of *Citrus* fruit classification can be significantly improved. For example, Bhargava et al., extracted color, statistical, texture, and geometric features from images to achieve a maximum detection accuracy of 98.48% for oranges and three other fruits [110]. Further, a Multilayer Perceptron (MLP) model combined with feature fusion techniques achieved an accuracy of 98.14% in classifying eight *Citrus* fruit varieties [111]. These advancements are critical for promoting the automation and intelligence of sorting, grading, and packaging processes in the *Citrus* processing industry. Table 1 presents the preprocessing techniques, feature selection and extraction methods, modeling techniques, and their optimal performance for these computer vision and spectroscopy technologies in *Citrus* quality assessment.

#### 5.1.2. *Citrus* Internal Quality Detection

Consumer satisfaction with *Citrus* fruits is influenced not only by their external appearance but also by their internal quality and flavor. *Citrus* fruits are rich in various nutrients, including carbohydrates, organic acids, vitamins, and flavonoids [3,146]. Key indicators for assessing the internal quality of *Citrus* fruits include SSC, TA, and the Brix–acid ratio [147]. Through spectral data analysis, researchers can non-destructively detect these components, enhancing industry competitiveness and profitability [132].

SSC plays a crucial role in determining the sweetness and overall nutritional value of *Citrus* fruits [35]. Traditional detection methods, such as refractometers, are often inefficient and may damage the samples, which has led to an increasing preference for spectral analysis [148]. For example, NIR spectroscopy has been used for SSC detection [63]. Tian et al. developed an effective predictive model for SSC using a portable Vis/NIR spectrometer and SNV-SPA-PLS algorithm [113]. Luo et al., applied HSI to predict SSC in “Nanfeng” mandarin, utilizing preprocessing techniques like MSC and SG, followed by modeling with PLSR and least-squares support vector machine (LSSVM) [35]. Their BOSS-CARS-PLSR model, which combined various wavelength selection techniques, achieved a coefficient of determination of 0.9376, demonstrating excellent predictive performance. Feature-level data fusion has also made significant advancements in the detection of internal quality. For example, Xu et al. explored the optimal combination of techniques for measuring total soluble solid content (TSSC) in “Luogang” oranges by integrating Vis-NIR spectroscopy, near-infrared spectroscopy, computer vision, and electronic nose technologies [112]. Through preprocessing and feature fusion using techniques such as SG, GA, mutual information fusion (MIF), and CNN, followed by PLSR modeling, the results showed that the fusion of Vis/NIR spectroscopy and computer vision was the optimal strategy for TSSC detection, significantly enhancing accuracy over traditional single-detection methods. This approach provides valuable insights into the internal quality detection of other fruits as well.

The unique flavor profile of *Citrus* fruits stems from the sweetness provided by their abundant sugars (including fructose and glucose), the acidity imparted by citric and malic acids, and the distinctive aroma conferred by volatile compounds. Furthermore, these fruits encompass plant-based chemicals such as alkaloids and flavonoids, which introduce subtle notes of bitterness and astringency [2]. Volatile compounds are the primary source of *Citrus*’s enticing aroma [5]. In flavor assessment, Serna-Escolano et al., successfully predicted the total soluble solids (TSS) and TA levels of “Fino” lemons using NIR spectroscopy and PLSR models [11]. Kim et al., developed predictive models for sugar content across multiple *Citrus* varieties using Vis/NIR technology [114], while Zeb’s team explored short-wave NIR spectroscopy for classifying sweetness [116].

It is noteworthy that NIR spectroscopy, while widely used, has some limitations in spectral resolution compared to MIR spectroscopy. Studies on the vitamin C, citric acid, total sugars, and reducing sugar content in “Valencia” oranges demonstrated that the MIR-PLSR model exhibited superior correlation and lower error compared to NIR spectroscopy, underscoring the advantages of MIR in detecting specific components [118]. Additionally, hyperspectral imaging technology in the NIR range has been effectively used for the rapid and quantitative detection of sugars, vitamin C, and organic acids in pomelo fruits [33]. Sabzi et al. combined computer vision systems, artificial neural networks (ANNs), and particle swarm optimization (PSO) techniques to predict the pH levels of *Citrus* fruits, showcasing the potential of integrating multiple technologies for internal quality assessment [40]. Fourier transform (FT) and PCA have proven robust for data preprocessing and discrimination in internal quality analysis. Gedikoğlu et al. employed FT and PCA with infrared spectroscopy to evaluate polyphenol and flavonoid content, as well as antioxidant activity, in *Citrus* fibers [65]. Similarly, terahertz spectroscopy has shown potential in detecting flavonoids, with Feng et al. demonstrating the relationship between the concentrations of hesperidin and naringin and terahertz spectra using PLSR [88]. The predictive models for these flavonoids achieved coefficients of determination of 0.99 and 0.97, respectively, surpassing the precision of NIR hyperspectral measurements [41].

Vitamin C, a vital nutrient in *Citrus* fruits, is commonly detected using traditional methods, such as titration, fluorescence, and high-performance liquid chromatography (HPLC). However, these methods are limited in accuracy and require chemical reagents [64]. Santos’s team successfully employed NIR spectroscopy to predict vitamin C content in various *Citrus* fruits, achieving correlation coefficients between 0.77 and 0.86, indicating promising potential for further research [64].

Moisture loss is a critical factor influencing *Citrus* fruit quality. Xu’s team applied Vis/NIR spectroscopy for post-harvest moisture detection in “Shatian” pomelos [115]. By using SG and MSC for preprocessing, along with GA for feature selection and PLSR for modeling, they achieved high accuracy in moisture content detection (R^2^ value of 0.712 and RMSE of 0.0488 in the validation set). Another study emphasized that fluctuations in moisture content, SSC, and TA could play a significant role in the granulation process of fruits, providing important information for improving fruit quality control and optimizing storage practices [117].

NMR spectroscopy has proven to be a powerful tool in *Citrus* fruit analysis. The Villa-Ruano team identified 35 metabolites through NMR metabolomics, emphasizing key amino acids, sugars, and organic acids as differential metabolites among *Citrus* varieties [119]. Similarly, Migues and colleagues assessed changes in chemical composition at various harvest stages, offering valuable information for *Citrus* breeding and providing essential support for juice quality control [95].

#### 5.1.3. *Citrus* Physicochemical Quality Detection

The quality of *Citrus* fruits is generally determined by a combination of various physical and chemical parameters, including color, shape, size, texture, SSC, and TA [149]. By integrating these parameters, a comprehensive evaluation of *Citrus* fruit quality can be achieved. The rapid advancement of microelectromechanical system (MEMS) technology has accelerated the adoption of non-destructive testing methods, leading to an increase in the popularity of portable testing instruments for on-site quality assessment.

Srivastava et al. developed a low-cost, portable handheld machine vision system that integrates seamlessly with smartphones, facilitating efficient data visualization and storage [120]. Through a smartphone application, the system enables real-time analysis of various *Citrus* quality parameters, such as chlorophyll content, sugar content, TSS, weight, pH, and volume. This portable system allows for immediate predictions and monitoring in the field, enhancing the flexibility and accessibility of *Citrus* quality evaluation.

Additionally, Srivastava’s team developed a smartphone-based portable spectrometer that utilizes UV-Vis-NIR spectroscopy to perform rapid, non-destructive testing of *Citrus* quality parameters and predict the attributes mentioned above [121]. In further research, Srivastava et al. investigated the use of four non-destructive sensing technologies—machine vision, UV-Vis-NIR spectroscopy, ultrasound, and electronic nose—integrating high-level, medium-level, and low-level data fusion techniques for analysis [122]. Specifically, high-level fusion employed a DT algorithm to combine results from multiple sensor technologies, enabling accurate predictions of key fruit quality parameters. In medium-level fusion, chlorophyll content and volume were predicted, while low-level fusion was applied to predict TSS, pH, and weight. This hybrid data fusion model demonstrated exceptional performance in predicting a range of quality parameters. It not only provided accurate assessments of *Citrus* fruit quality but also showed considerable potential for supporting harvest decision-making, thus underscoring the critical role of integrating multidisciplinary sensor technologies. This integration enhances both the efficiency and scope of quality detection in *Citrus* fruits.

#### 5.1.4. *Citrus* Quality-Based Ripening and Harvesting Detection

Determining the maturity of *Citrus* fruits is crucial for optimal harvesting, storage, and sales, yet it remains a significant challenge in *Citrus* quality assessment [8]. The Brix–acid ratio, defined as the ratio of SSC to TA, is an important indicator for evaluating the maturity of *Citrus* fruits [150]. As fruits ripen, both their physical properties and chemical indicators undergo substantial changes. Therefore, selecting the ideal harvest time requires a comprehensive evaluation of maturity indices, external fruit characteristics, and market demand, among other factors [123,128,129].

The *Citrus* peel color is widely used for maturity assessment due to its practical acceptance and ease of application. Zakiyyah et al. utilized color indices in conjunction with a SVM model to predict *Citrus* maturity, achieving an accuracy of 88.71% [123]. Additionally, transfer learning, a powerful machine-learning strategy, can significantly enhance model efficiency and generalization. By leveraging transfer learning, a *Citrus* maturity prediction accuracy of over 96% was achieved even with a small sample size [124]. This approach offers new insights and technical support for the ongoing monitoring of *Citrus* maturity.

In addition to peel color, changes in the internal chemical composition of *Citrus* fruits are pivotal for determining maturity [11]. For example, the Pires team used short-wave NIR reflection spectroscopy to perform non-destructive evaluations of internal quality attributes in Ortanique *Citrus*, developing predictive models for parameters such as pH, SSC, TA, and the maturity index (MI) [129]. Furthermore, as the internal composition of *Citrus* changes, its fluorescence spectral characteristics also shift. Combining fluorescence spectroscopy with CNN, the fluorescence values of *Citrus* peel were used to estimate the Brix–acid ratio in the fruit pulp, achieving an absolute prediction error of just 2.48, surpassing the accuracy of traditional methods [83].

The fusion of data from multiple non-destructive testing technologies can further improve prediction accuracy. For instance, Riza et al. combined Vis-NIR reflectance spectroscopy with fluorescence spectroscopy and applied data fusion techniques, resulting in a maturity model with a determination coefficient of 0.91—significantly higher than using either spectrum alone [84]. Additionally, by overlaying reflectance images with fluorescence images into a six-channel input array and integrating this with a DCNN regression model, Riza and colleagues improved detection accuracy [130]. Sandra et al. also developed a sensor data fusion method combining Vis-NIR and fluorescence spectroscopy with ANN, successfully achieving precise detection of key parameters such as TSS, acidity, hardness, and maturity in Pontianak Siam oranges [131].

In precision agriculture, accurately identifying fruit maturity while the fruit is still on the tree is essential for efficient management. Liu et al. developed a machine vision algorithm based on the elliptical boundary model for *Citrus* maturity detection [125], while Chen et al. used CNN and visual saliency maps to detect maturity with high accuracy [126]. To overcome challenges like uneven lighting and fruit occlusion in orchard environments, Chen and colleagues integrated an improved Hough transform with deep-learning techniques to enhance accuracy [24]. Additionally, estimating *Citrus* yield is crucial for growers to maintain competitiveness and make informed decisions. Apolo-Apolo et al. employed drone UAV imagery combined with deep learning to accurately predict the fruit size and total yield of individual *Citrus* trees [127].

Predicting freshness is equally important in the storage, transportation, and wholesale management of *Citrus* fruits. Traditional visual inspection methods often produce significant inaccuracies. Yu et al. explored a method that combines visible-light imaging with CNN to predict *Citrus* freshness, achieving an impressive prediction accuracy of 95.6%. This approach offers strong technical support for more refined management of the fruit market [128].

### 5.2. Citrus Damage Detection

*Citrus* damage encompasses both physical and biological damages that affect the fruit’s quality, marketability, and safety [137]. Physical damage includes issues like cracks, bruises, and frost damage, which compromise the fruit’s appearance and integrity. Biological damage caused by bacteria, fungi, and other microorganisms leads to lesions, rot, and other quality-degrading issues. Consequently, effective damage detection is crucial for maintaining *Citrus* fruit quality and ensuring food safety.

#### 5.2.1. *Citrus* Defect Detection

NDT techniques play a vital role in detecting physical defects in *Citrus* fruits, such as spots, cracks, shape abnormalities, bruises, granulation, and frost damage [8]. These defects not only affect the visual appeal of the fruit but may also influence its internal quality and storage life [133]. Early defect detection in the *Citrus* supply chain is, therefore, essential for minimizing post-harvest losses and improving product quality [3].

In recent years, CNN-based NDT technologies have made significant strides. Jahanbakhshi et al. introduced a sparse random pooling technique to enhance CNN model performance, achieving 100% accuracy in classifying acid lemon images [25]. Additionally, the Yolo-FD detection model proposed by Lu et al., combining PSO with extreme learning machine (ELM), performed exceptionally well in defect classification, with a mean average precision (mAP) improvement of 1.4% over YOLOv5 and a classification accuracy of 91.42% [10]. To enhance detection accuracy for latent skin defects, fluorescence imaging, coupled with the CBAM attention mechanism and DIoU loss function, was applied to optimize the YOLOv5 model, achieving better performance than YOLOv5x in terms of mAP, precision, and recall [133]. These advancements showcase the potential of CNN models in precisely identifying defects in *Citrus* fruits.

To address the challenges posed by lengthy hyperspectral image acquisition and analysis, Zhang et al., developed a multispectral image classification algorithm based on Vis/NIR hyperspectral imaging. By applying PCA for dimensionality reduction and selecting characteristic wavelengths, they achieved efficient detection of four common *Citrus* defects, with a classification accuracy of 96.63% [4].

*Citrus* granulation, a physiological issue caused by water deficiency or uneven moisture distribution, can also be detected using advanced imaging technologies. Jie et al. employed hyperspectral imaging and introduced batch normalization into a CNN model, achieving 100% accuracy in the training set [38]. Additionally, data fusion techniques can enhance detection accuracy for both *Citrus* quality and physiological diseases. For thick-skinned fruits like pomelos, internal quality prediction using transmission spectroscopy alone is often less accurate [132]. To improve prediction performance, Sun et al. integrated Vis/NIR transmission spectroscopy with CV technology and applied feature-level fusion. By extracting principal components from preprocessed spectral data and external features from machine vision, this approach successfully detected and estimated internal granulation issues, achieving 99% accuracy with the PCA-GRNN model, far outperforming traditional near-infrared methods [117,134].

Frost damage, which causes dehydration of the fruit pulp and potentially leads to bitterness and nutrient loss, is another critical issue for *Citrus* quality [66]. Tian et al. applied Vis/NIR transmission spectroscopy in combination with a deep One-Dimensional Convolutional Neural Network (1D-CNN) for online detection of early-stage frost damage in *Citrus* fruits. The method achieved an overall detection accuracy of 91.96%, demonstrating its effectiveness for early detection of frost-induced damage [66].

#### 5.2.2. *Citrus* Disease Detection

*Citrus* diseases can be classified into three main categories based on their pathogens: bacterial, fungal, and viral diseases. Among the most common and impactful are HLB, Canker, Black Spot, Scab, and Anthracnose, which significantly affect *Citrus* growth and yield [15,103].

HLB, also known as *Citrus* greening disease, is caused by a bacterial pathogen and is particularly devastating. It leads to spots, pseudo-melanin deposition, and green areas on the fruit surface while weakening the immune system of the *Citrus* tree, making it more susceptible to other diseases and potentially causing premature fruit drop [79,135]. This disease poses a significant threat to the *Citrus* industry. Traditional detection methods, such as PCR and antibody-based tests, have limitations, including high costs, time-consumption, destructiveness, and insufficient sensitivity [79]. To overcome these limitations, researchers have developed a range of novel detection methods. For example, Lan et al. integrated drone-based remote sensing with multispectral imaging, employing machine-learning algorithms that achieved detection accuracies of 100% and 97.28% for HLB, demonstrating the effectiveness of this approach [53]. Sanchez and colleagues combined handheld Raman spectroscopy with chemometric analysis to distinguish between healthy, HLB-infected, and nutrient-deficient *Citrus* trees, with detection accuracies of 98% for grapefruit and 87% for oranges [79]. Xu et al. developed a computer vision system with reflection and transmission modes to detect HLB symptoms in *Citrus* leaves, achieving 96.67% accuracy in reflection mode and 88.33% in transmission mode [135]. Additionally, He et al., developed a handheld device integrating multi-color fluorescence and multispectral reflectance imaging technologies, achieving a detection accuracy of 96.5% for HLB by feeding fused image data into the MobileNetV3 model [142]. These studies highlight the potential of combining non-destructive detection methods with machine learning to rapidly and accurately detect HLB.

*Citrus* Canker, marked by lesions with water-soaked brown edges and yellow halos on stems and fruits, demands early detection to optimize pesticide use [44]. Le et al. proposed the CitrusNet model, based on deep learning, and optimized using the Squeeze-and-Excitation (SE) block algorithm, achieving a detection accuracy of 92.33% for *Citrus* Canker [28]. Additionally, studies using hyperspectral imaging technology and Radial Basis Function (RBF) algorithms demonstrated the effective use of moisture indices and the modified chlorophyll absorption reflectance index for detecting *Citrus* Canker [44].

*Citrus* Black Rot, characterized by Black Spots on the fruit’s surface, can also be detected using advanced imaging techniques. Ghooshkhaneh et al., employed visible and near-infrared reflectance spectroscopy to detect Black Rot disease in oranges, revealing that the disease tends to develop more frequently at the bottom of the fruit [69]. Furthermore, an optimized CNN model combined with machine vision techniques has significantly enhanced the detection accuracy of *Citrus* Black Spot disease [29]. Hyperspectral imaging, paired with PLS analysis and the KNN model, has enabled precise identification of different stages of *Citrus* Black Spot disease [138].

Fungal pathogens, such as *Penicillium digitatum*, which causes green mold disease, are another significant threat to *Citrus* [151]. If not promptly addressed, decayed fruits can lead to substantial economic losses [137]. Chakraborty et al., proposed a CNN-based MobileNetV2 architecture, successfully detecting freshness and decay defects in oranges with a validation accuracy of 99.61% [30]. As early-decaying *Citrus* fruits often resemble healthy ones, traditional visual inspection methods are often inaccurate and time-consuming [139]. To overcome these limitations, researchers have developed a range of non-destructive detection methods. For example, Li et al. combined portable near-infrared diffuse reflectance spectroscopy with chemometrics, using algorithms such as SIMCA, SVM, and PLS-DA to achieve 100% accuracy in detecting early-stage *Citrus* decay [67]. Moreover, multispectral and hyperspectral imaging technologies have shown great promise in *Citrus* rot detection. Li et al., proposed an algorithm integrating multispectral principal component images, two-dimensional empirical mode decomposition, and an improved watershed segmentation method, achieving accuracies of 97.3% for decayed and 100% for healthy fruits [140]. Additionally, the dual-wavelength image detection technique, which combines spectral classification and image processing in hyperspectral imaging, enables early-stage detection of rotting oranges, achieving an overall classification accuracy of 96.6% [139]. Luo’s team developed a multispectral classification algorithm based on Vis-NIR hyperspectral imaging, optimizing spectral variables with PCA to select four characteristic wavelengths, achieving a classification accuracy of 98.6% [141]. Structural Illumination Reflectance Imaging (SIRI) technology has shown potential in early decay detection. By combining real-time (RT) images with a CNN model, the overall classification accuracy for early decay detection in four types of *Citrus* fruits reached 90.6% [137]. These studies highlight the effectiveness of *Citrus* decay detection technologies, offering strong support for early diagnosis and management of *Citrus* diseases.

To address the challenge of identifying surface defects related to *Citrus* diseases, Tan et al. developed an ABC-SVM-based *Citrus* surface defect identification algorithm, achieving an average recognition rate of 98.45% for defects such as Scab and Anthracnose [27]. Kukreja et al. also developed a CNN-based algorithm for detecting visible *Citrus* defects, achieving a detection accuracy of 89.1% [103]. Furthermore, a method combining Duck Optimization Algorithm (DOA) with Capsule Network (ECN)-enhanced Deep-Stacked Autoencoder (DSSAE) models, known as DOECN-CDDCM, achieved a classification accuracy of 98.4% for *Citrus* disease detection and classification [136]. To improve accuracy further, Dhiman’s team fused data from both NIR and RGB sensors, employing data-layer fusion and decision-layer fusion techniques, which significantly enhanced the model’s ability to identify multiple diseases [143].

With the continuous optimization of algorithms and the growing adoption of detection equipment, the automated detection and management of *Citrus* diseases are set to drive further modernization and intelligence in the *Citrus* industry.

### 5.3. Citrus Adulteration and Traceability Detection

One of the significant challenges in quality control within the food industry is contamination and fraud, particularly concerning the product adulteration and falsification of production origins [49]. *Citrus* juice adulteration and commercial fraud, such as the addition of water, low-cost juices, and excessive use of food additives, have become major concerns in the *Citrus* industry, particularly as consumer demand for fresh, safe, healthy, and high-quality food continues to grow [94]. To address these issues, various analytical techniques have been developed to verify the authenticity of *Citrus* juice. For example, Mohammadian et al. used FT-IR spectroscopy to assess the vitamin C content in lime juice, achieving an accuracy rate of 96% in detecting its authenticity [145]. Furthermore, with the rise of mixed fruit juices, the detection of added water or cheaper juices has become increasingly important. To address this, Marchetti et al. combined proton nuclear magnetic resonance (^1^H NMR) with PLS analysis to determine the relative percentage of pure juice in mixed fruit juices, providing an efficient and reliable solution for juice adulteration detection [94].

Traceability is especially critical in the *Citrus* industry, as the production region directly influences the unique sensory characteristics of *Citrus* fruits, which in turn affect consumer preferences and the commercial value of the product [92]. However, these differences are often difficult to discern with the naked eye. Advanced NDT technologies have been employed to address this challenge and ensure accurate identification. For instance, Ruggiero et al. used reflection NIR spectroscopy combined with chemometrics to assess the quality characteristics of Italian lemons, effectively distinguishing different lemon varieties and their origins [144]. Similarly, Lin et al. used NMR spectroscopy coupled with chemometric methods to identify and quantify 62 components from sweet oranges grown in four major *Citrus*-producing regions in China [92]. Salazar and colleagues employed NMR spectral analysis to rapidly and accurately determine the geographical origins of orange juice from various regions in Argentina [93]. These studies demonstrate the potential of NDT methods for ensuring the authenticity of *Citrus* products, which is essential for quality control and market regulation.

These advancements in NDT technologies have significantly enhanced market transparency, ensuring the authenticity of *Citrus* products and promoting the healthy development of the *Citrus* industry.

## 6. Conclusions and Future Trends

This review examines the principles and applications of computer vision and spectroscopy technologies for *Citrus* fruit quality assessment. It covers a range of technologies, including traditional CV, HSI, and MSI, as well as spectral methods such as IR, Raman, fluorescence, THz, and NMR. These technologies have significantly advanced the *Citrus* industry, owing to their non-destructive nature, efficiency, cost-effectiveness, and reliable performance. Each of these technologies possesses unique characteristics, offering robust tools for the comprehensive evaluation of *Citrus* quality. Table 2 summarizes the detection characteristics, advantages, and limitations of these various computer vision and spectral technologies for *Citrus* fruit quality assessment.

Traditional CV techniques are effective at capturing the external quality features of *Citrus* fruits. However, they face multiple challenges, including high dimensionality and redundancy in feature extraction, interference from external light, and a high dependence on camera characteristics, all of which directly affect the overall efficiency of the system [152]. To address these challenges, researchers have explored multi-image-processing techniques to construct 3D views of *Citrus* samples on trees and have investigated various intelligent image-processing algorithms to enhance data-processing capabilities and mitigate the impact of changes in camera performance on detection results [22]. However, traditional CV techniques often fail to achieve ideal detection accuracy when dealing with defects that have low contrast or are difficult to detect externally, and they are unable to assess the internal quality of *Citrus* [153]. In contrast, HSI and MSI technologies, which incorporate spectral data, offer a more comprehensive approach by capturing both external features and providing detailed insights into internal characteristics. These technologies significantly improve detection accuracy, enabling more thorough and holistic quality evaluations. However, HSI technology requires substantial computational resources and time, and it incurs high costs, thus hindering its widespread adoption in commercial settings [154]. Compared to HSI, MSI offers faster detection speeds and lower equipment costs but with lower accuracy and limited information for certain specific tasks, making it more suitable for applications that require spectral information without the need for fine details.

Spectral technologies offer rapid, non-destructive methods for evaluating the internal quality of *Citrus* fruits. Techniques such as IR Spectroscopy, Raman Spectroscopy, fluorescence spectroscopy, THz, and nuclear magnetic resonance NMR have demonstrated excellent detection capabilities, each with unique strengths and applications. IR spectroscopy is the most commonly used technique for efficiently analyzing a range of chemical components in *Citrus*, including sugars and acidity, without damaging the sample. Among these, NIR spectroscopy is more commonly used; however, the MIR region provides clearer results. In Borba’s experiment, MIR demonstrated superior performance in detecting vitamin C and citric acid [118]. However, the complex nature of its spectra necessitates the use of advanced chemometric methods for accurate identification. For thick-skinned fruits like grapefruit, the prediction accuracy of internal quality attributes using transmission spectroscopy is lower than that of reflection spectroscopy [132]. Raman spectroscopy has the advantage of high sensitivity, and SERC technology can detect low-concentration substances. However, it is easily interfered with by fluorescence, and the high cost of Raman spectrometers limits its application in the *Citrus* industry. Fluorescence spectroscopy offers fast detection and high sensitivity. However, it can only detect substances with fluorescent properties, thus limiting its application in the *Citrus* industry. THz spectroscopy has shown potential in detecting flavonoids [88], but it is limited by its cost. Its application in the *Citrus* industry requires further research and development. Despite the drawbacks of high cost and complex operation, NMR is still regarded as a convenient and non-invasive method in terms of sample measurement, preparation, recovery, and analysis time, compared to the application of mass spectrometry (MS) in *Citrus* fruits metabolomics [61]. For large-scale application in the *Citrus* industry, however, this remains challenging. It is primarily used in laboratory settings to analyze various chemical components, pesticide residues, and product traceability in *Citrus*. However, these spectral technologies enable detailed analysis of internal fruit components, including sugars, acidity, and nutrient content, providing valuable insights for quality control and disease detection. However, these technologies have limitations in assessing external quality characteristics, such as color, size, and visible defects, leading to incomplete evaluations when used alone.

**Figure 3:**
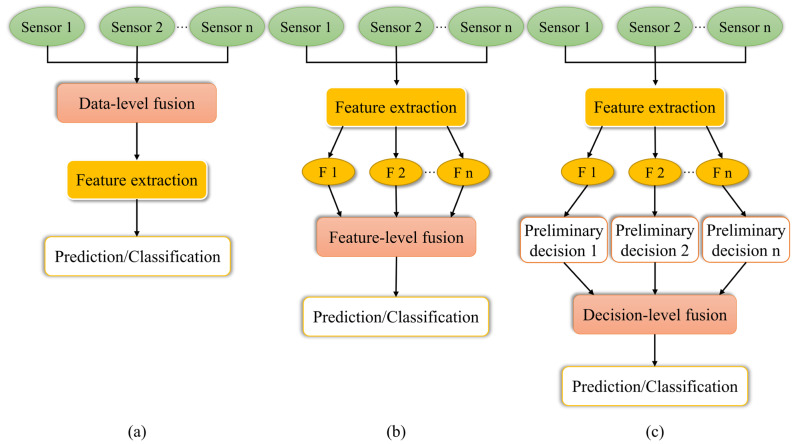
Data fusion levels: (**a**) data level, (**b**) feature level, and (**c**) decision level.

When compared to relying solely on computer vision for external feature analysis or spectroscopy for internal quality detection, multi-sensor data fusion brings about a transformative advancement [112]. This methodology significantly enhances the accuracy and robustness of detection systems, expands the measurement scope, and provides comprehensive and robust support for *Citrus* fruit sorting and grading systems. It effectively addresses the limitations of single-sensor detection methods, enabling more precise evaluations in complex scenarios [62]. By integrating data from multiple sensors and leveraging their complementary and synergistic effects, along with chemometric methods, a more comprehensive *Citrus* quality assessment can be achieved [155,156,157]. Multi-sensor data fusion models can be categorized into three main levels: data-level fusion, feature-level fusion, and decision-level fusion [156,158], as illustrated in Figure 3. Specifically, data-level fusion not only enhances both accuracy and comprehensiveness [84,130,131,159] but also allows for maximum retention of detailed information [160]. However, it comes with high computational and storage costs, and sensitivity to fluctuations in sensor performance. Feature-level fusion reduces noise and improves data quality while preserving detail, thus enhancing processing efficiency and accuracy [112,134]. However, testing various feature extraction and preprocessing combinations can be cumbersome and computationally expensive [161]. Decision-level fusion has the advantages of real-time performance, low communication overhead, and high fault tolerance, enabling reasonable decisions even in the event of sensor failures. However, it is highly algorithm-dependent, may lead to information loss, requires complex preprocessing, and results in significant computational overhead. Despite challenges such as complex data processing, high computational resource demands, sensor synchronization issues, and data inconsistencies, data fusion technology still holds substantial potential for further development.

These technologies not only provide critical technical support for quality control and production efficiency enhancement in the *Citrus* industry but also play a pivotal role in advancing agricultural intelligence and precision. While *Citrus* computer vision and spectral detection technologies show promising prospects, further development is required in the following areas:

First, there is a strong need for deeper technological convergence and innovation. This requires close collaboration between interdisciplinary teams to seamlessly integrate cutting-edge technologies, such as computer vision, spectral detection, and artificial intelligence. Developing more efficient and accurate inspection systems through multi-sensor data fusion techniques, which leverage the complementary strengths of various NDT technologies, is essential. Intelligent data fusion approaches, combining advanced AI-based modeling strategies with innovative drift compensation techniques, hold great promise in overcoming existing bottlenecks and challenges [152]. Despite the potential of this direction, comprehensive application cases remain scarce. There is an urgent need for increased research and development efforts, particularly through collaboration between academia and industry, to foster the growth of this field.

Second, the rapid acceleration of intelligence and automation is crucial. With the growing prevalence of IoT, big data, and artificial intelligence techniques, the *Citrus* inspection sector is advancing toward a new stage of intelligence and automation, aiming to improve detection accuracy while achieving smarter detection. For example, to address the challenges posed by the color similarity between green *Citrus* fruits and their background, Zheng et al. enhanced detection accuracy by applying artificial intelligence techniques. They proposed the YOLO BP algorithm, which achieved an accuracy of 86%, providing strong support for the detection of green *Citrus* fruits [162]. However, in the field of chemometrics, there remains an overreliance on traditional experimental trial-and-error methods in the pursuit of optimal preprocessing methodologies, which are both inefficient and costly. Furthermore, HSI data collected contain multiple spectral bands, which exhibit significant redundancy and collinearity [163]. In hyperspectral image data processing, the challenge of quickly determining the optimal number of bands for grading persists, often requiring extensive comparative studies to identify the best band combinations [35]. Therefore, there is an urgent need to replace manual experimentation with artificial intelligence techniques, developing scientific preprocessing selection and integration algorithms to optimize detection procedures, enhance overall performance, and lay a solid foundation for the intelligent and automated advancement of *Citrus* detection technology.

Lastly, cost reduction and broader adoption are crucial. Through technological innovation and large-scale production, the cost of testing equipment can be reduced, making it more accessible to the majority of farmers and enterprises. The development of suitable handheld imaging instruments and portable spectrometers that meet the requirements of real-time fruit quality monitoring is vital. These portable devices are characterized by their low cost, wide applicability, compact size, minimal sample preparation requirements, and high sensitivity and precision, thereby supporting rapid fruit quality evaluation. However, the development of handheld instruments based on multi-sensor fusion technology remains limited. There is a need for significant investment in the development of multifunctional detection equipment based on data fusion technologies to promote the widespread adoption and dissemination of such tools [122].

## Figures and Tables

**Table 1:** Application of computer vision and spectroscopy in *Citrus* fruits’ quality assessment.

Detection Technology	Sample	Measurement Properties	Preprocessing	Feature Selection and Extraction	Modeling Technology	Best Performance	Reference
Computer vision	Three kinds of bergamots	Peel color, dimensional features, and hardness	White balance, color correction, standardization	RGB to Hunter L, a, b, shape, PCA	LDA	Accuracy = 80.49%	[20]
Computer vision	Pontianak Siam oranges	Fruity flavor	Digitization	RGB	KNN	Accuracy = 80%	[23]
Computer vision	Oranges	Color and sweetness	Contrast, sharpening, smoothing, edge detection, filtering	RGB	KNN, DT, SVM, Neural Network, LR	LR: accuracy = 97%	[105]
Computer vision	Oranges	Size measuring	Data augmentation, find contour, crop, resize, median filter	Binary image, CNN, Cycle GAN, RGB, HSV, YCrCb, contours	YOLOv5, PLS	Accuracy = 95.6%, the overall error = 10.12%	[106]
Computer vision	Oranges and other fruits	Size and maturity	OTSU, voxel mapping, projection matrix estimation	RGB to HSV, contours, 3D reconstruction, volume conversion	FRBC	Classification accuracy = 98.5%	[22]
Computer vision	Sweet lime fruit	Weight	Median filter, grayscale conversion, OTSU, binarization	Canny, 1D, 2D	DA, NR, FFANN	R^2^ = 0.9931, MAPE = 2.306%	[21]
Computer vision	Sweet lime fruit	Weight	Channel separation, median filter, grayscale, OTSU	1D, 2D	SVM, GA-ANFIS, PSO-ANFIS	R^2^_p_ = 0.9536, RMSEP = 4.3113	[107]
Computer vision	*Citrus* fruits	Surface feature and weight	Image resizing	-	SortNet	Classification accuracy = 97%, grader accuracy = 91.3%	[9]
Computer vision	*Citrus* fruits	Fruit segmentation, color, and size classification	Histogram equalization, rotation, zoom	-	DT	Segmentation accuracy = 97%, color accuracy = 94%, size accuracy = 90%	[109]
Computer vision	Oranges, avocados, bananas and apples	Grading and classification	Background separation, image scaling, Gaussian filtering, fuzzy segmentation	Color, statistical, texture, geometric features	KNN, SVM, SRC, ANN	Accuracy = 98.48%	[110]
Computer vision	Grapefruit, Moussami, Malta, lemon, Kinnow, Local lemon, Fuetrells, and Malta Shakri	Classification	ROI, Binary, histogram, texture, spectral, data augmentation	CFS	MLP, RF, J48 and Naive Bayes	MLP: accuracy = 98.14%	[111]
Computer vision	Bam, Blood, and Thomson orange	pH	Threshold segmentation	Color, texture, histogram, moments, shape	ANN-PSO, MLP	Bam: R^2^ = 0.950, Blood: R^2^ = 0.935, Thomson: R^2^ = 0.957	[40]
HSI	Nanfeng mandarin	SSC	SGS-MSC	BOSS, CARS, IRIV	PLSR, LSSVM	R^2^_p_ = 0.9376, RMSEP = 0.3986	[35]
HSI	Pomelo	Sugar, vitamin C, organic acid	ROI	-	RBF-PLS	Sugar: R^2^_T_ = 0.872, RMSET = 1.404%; Vitamin C: R^2^_T_ = 0.872, RMSET = 61.540 mg/kg; organic acid: R^2^_T_ = 0.866, RMSET = 1.573 g/kg	[33]
HSI	Pomelo fruits	Naringin content	SG	-	PLS	R^2^_CV_ = 0.933, RMSECV = 0.345	[41]
VIS/NIR, computer vision, electronic nose	“Luogang” Orange	TSSC, and water content	SG	GA	CNN-PLSR	TSSC: R^2^ = 0.8580, RMSE = 0.4276; water content: R^2^ = 0.7013, RMSE = 0.0063	[112]
Vis/NIR	“Gannan” navel orange	SSC	Smoothing, MSC, SNV, 1D	SPA, CARS, GA	PLS	R^2^_p_ = 0.9165, RMSEP = 0.5684	[113]
Vis/NIR	Unshiu, Cheonhyehyang, Hallabong	Sugar content	MSC, SNV, SG, MM	-	PLSR, VIP-PLSR, Full-ANN, PCA-ANN, PLS-ANN, 1D-CNN, Ensemble Type-1, 2, 3, 4	R^2^_T_ = 0.839, RMSET = 0.516	[114]
Vis/NIR	“Shatian” pomelo	Water content and granulation degree	1D, SR, LM, IM, SG, MSC	RCA, MI-SPA, GA, PCA, LDA	PLSR	R^2^_v_ = 0.712, RMSEV = 0.0488; accuracy = 100%	[115]
Vis/NIR	Pomelo	SSC	SNV, MSC, 2D	CARS, SPA, PCA	PLSR, SVR	R^2^_v_ = 0.85, RMSE = 0.98	[63]
NIR	“Fino” lemons	TSS, and TA	MSC	Spectral conversion	PLS-R, PLS-DA	TSS: R^2^ = 0.84, RMSEP = 0.42; TA: R^2^ = 0.72, RMSEP = 0.45	[11]
NIR	Red Blood, Mosambi, and Succari oranges	Brix, TA, Brix: TA, BrimA, and sweetness classification	SG	PCA	PLSR, Tree, Ensemble, KNN, LDA, SVM	Brix: R^2^ = 0.57, TA: R^2^ = 0.73, Brix: TA: R^2^ = 0.66, BrimA: R^2^ = 0.55, classification accuracy = 80.03%	[116]
NIR	*Citrus* fiber	Total polyphenol, total flavonoid, oxygen radical absorbance capacity values, and the pH	Fixed block mean, polynomial subtract (1st order), smoothing	PCA	GLM	R^2^ = 0.96	[65]
NIR	Oranges, lemons, clementines, tangerines, and Tahiti limes	Ascorbic acid, dehydroascorbic acid, total vitamin C, soluble solids, total acidity, and juiciness	SNV, SG, 1D, 2D, MSC, normalization	PCA	LDA, PLSR	Vitamin C: R^2^ = 0.77–0.86	[64]
NIR	“Sai Num Pung” tangerine fruit	MC, SSC, TA, and granulation rate	SNV, MSC, normalization, derivatives	PCA	PLS, LDA, QDA, PLS-DA, KNN, SSOM	Predictive ability = 93.7%, model stability = 95.3%, correctly classified = 94.0%	[117]
NIR, MIR	“Valencia” oranges	Vitamin C, citric acid, total and reducing sugar content	Mean center, SNV, SG, normalization	-	PLS	MIR models had lower prediction errors than NIR models	[118]
THz	Valencia sweet orange	Naringin, and hesperidin	MSC, SNV, 1D, 2D	-	PLSR	Naringin: R^2^ = 0.99, RMSEP = 2.97%; hesperidin: R^2^ = 0.97, RMSEP = 4.48%	[88]
NMR	Lemons, tangerines, oranges, and grapefruits	Specific amino acids, sugars, and organic acids	-	PCA	OPLS-DA	Valencia oranges had the highest concentration of ascorbic acid (>2 mM)	[119]
NMR	8 *Citrus* varieties grown in Uruguay	Sugar, citric acid	Zero padding, Fourier transform, phase correction, baseline correction, normalization	PCA	PLS-DA, OPLS-DA	Sweetening power/citric acid: R^2^ = 0.79	[95]
Computer vision	*Citrus*	Chlorophyll, sugar, TSS, pH, weight, volume	Grayscale, OTSU, morphological operations, watershed	Dominant color method, Color and texture characteristics	PCR, PLSR, MLR, ANN	Ch a: accuracy = 70.38%; Ch b: accuracy = 79.72%; TSS: accuracy = 78.94%; sugar: accuracy = 73.97%; weight: accuracy = 68.68%; volume: accuracy = 48.98%; pH: accuracy = 63.11%	[120]
UV-Vis-NIR	*Citrus*	Chlorophyll, sugar, TSS, pH, weight and volume	SNV, spectral average	PCA	ANN, MLP, PLSR, PCR	Ch a: accuracy = 76.71%; Ch b: accuracy = 82.86%; TSS: accuracy = 87.88%; sugar: accuracy = 77.33%; weight: accuracy = 62.47%; volume: accuracy = 18.98%; pH: accuracy = 80.64%	[121]
Computer vision, UV-Vis-NIR spectroscopy, ultrasound, and electronic nose	*Citrus* fruits	Chlorophyll, sugar, TSS, pH, weight and volume	Baseline correction, segmentation, noise elimination, amplitude and time of flight extraction, scaling and normalization, color and texture extraction, multiple to single spectrum conversion, attenuation and propagation delay conversion	PCA	Statistical modeling methods (MLR, PCR, PLSR) and Five Different ANN modeling methods	TSS accuracy = 95.64%; chlorophyll (Ch a accuracy = 96.78%, Ch b accuracy = 97.76%); sugar accuracy = 97.36%; pH accuracy = 78.31%; weight accuracy = 91.45%; Volume accuracy = 36.64%	[122]
Computer vision	Orange	Maturity	OTSU, histogram pattern, thresholding, binarization	RGB, L*, a*, b, HSV	LR, DT, RF, SVM	SVM: accuracy = 88.71%	[123]
Computer vision	Lemon	Maturity	Image resizing, filter, color space conversion, grayscale, OTSU	ROI	VGG, ResNet, DenseNet, NASNet Large, MobileNet, Inception V3	VGG: accuracy = 96.134%	[124]
Computer vision	Grapefruit	Maturity	RGB to Y’CbCr, elliptical boundary model segmentation, morphological operations	Color area selection, ellipse fitting, Douglas–Peucker algorithm	Polynomial Fitting	Total correct recognition rate = 93.5%	[125]
Computer vision	Tangerine	Maturity	Data augmentation	MSSS	YOLOv5, ResNet34	Accuracy = 95.07%	[126]
Computer vision	*Citrus*	Maturity	RGB, HIS, graying, OTSU, binarization, morphological operations	Area evaluation, Canny, corner detection, edge labeling algorithm, extract contour fragments, Hough transform	Morphological characteristics statistics	Accuracy = 97.44%	[24]
Computer vision	*Citrus* orchard	Fruit production, and fruit size	ROI, data augmentation	CNN	Faster R-CNN, LSTM	Estimate error = 7.22%	[127]
Computer vision	Ponkan mandarins	Freshness	Image masking, data augmentation	ResNet-18	CNN	Prediction accuracy = 95.6%	[128]
NIR	“Ortanique” *Citrus*	pH, SSC, TA, and MI	SNV, PSNV, MSC, Norris derivative, SPLINE, SG, CR	-	PLS	pH: R^2^ = 0.80; SSC: R^2^ = 0.79; TA: R^2^ = 0.73; MI: R^2^ = 0.69	[129]
Fluorescence spectroscopy	Satsuma mandarin	Brix–acid ratio, and maturity	-	-	CNN, PCR	Absolute error = 2.48	[83]
Vis-NIR, fluorescence spectroscopy	Mandarin Batu 55 oranges	SSC, TA, and maturity	MA, SG, SNV, MSC	PCA	PLSR	R^2^ = 0.91, RMSE = 2.4555	[84]
Computer vision, fluorescence imaging	Mandarin Batu 55 oranges	Maturity, SSC, acidity, firmness, and Brix–acid ratio	MA, SG, SNV, MSC	PCA	DCNN	Acidity: R^2^ = 0.83; Brix–acid ratio: R^2^ = 0.94; SSC: R^2^ = 0.86; firmness: R^2^ = 0.91	[130]
Vis-NIR, fluorescence spectroscopy	Pontianak Siam oranges	TSS, acidity, firmness, and maturity	MA, SG	PCA	ANN	TSS: R^2^ = 0.89; acidity: R^2^ = 0.96; firmness: R^2^ = 0.97; maturity: R^2^ = 0.99	[131]
Computer vision	Sour lemons	Defect	ROI, normalization, data augmentation	-	CNN, KNN, ANN, Fuzzy, SVM, DT	Accuracy = 100%	[25]
Computer vision	*Citrus* fruits	Peel defects, and fruit morphological characteristics	Image stitching, data augmentation	Image-processing technology	Yolo-FD, PSO-ELM	Yolo-FD: average accuracy = 98.7%; PSO-ELM: accuracy 91.42%, R^2^ = 0.9044, MSE = 0.8497	[10]
HSI	“GuanXiMiYou” *Citrus*	Granulation	Image correction, data augmentation	-	LS-SVM, BP-NN, CNN, CNN with batch normalization	Training set accuracy = 100%	[38]
HSI	*Citrus*	SSC, and TA	OTSU, MSC	CARS, SPA, CARS-SPA	PLS, MLR, LS-SVM	R^2^_p_ = 0.911, RMSEP = 0.4032	[132]
MSI	“Nanfeng” mandarins	Defects	Image calibration	PCA	Defect detection algorithm based on PC-2 image and ratio image combined with simple threshold method	Classification accuracy = 96.63%	[4]
Fluorescence imaging	*Citrus*	Epidermal defects	Mark, scale, crop and add noise	CBAM, FPN, PAN	YOLOv5	Map = 95.5%, precision = 94.0%, recall = 95.1%	[133]
Vis/NIR	“Orah” oranges	Freezing damage	DCM	CARS, SPA,	PLSDA, SVM, CNN	Overall accuracy = 91.96%	[66]
Vis/NIR, computer vision	Honey pomelos	SSC, TA, and moisture content	Normalization, SG, MSC	PCA	LDA, SVM, GRNN	Moving average = 0.9950, classification sensitivity = 0.9750, classification specificity = 0.9934	[134]
Computer vision	*Citrus* leaves	HLB	Threshold segmentation, connectivity analysis, morphology, fitted ellipse, affine transformed	GLCM, grayscale histogram	MLP, RF, LR	Reflection modes accuracy = 96.67%, transmission modes accuracy = 88.33%	[135]
Computer vision	Pomelo trees	Canker	-	-	*Citrus*Net, SVM	Accuracy = 92.33%	[28]
Computer vision	*Citrus*	Ripeness level, and Black Spot	Data augmentation, CAE	-	GoogleNet, ResNet18, ResNet50, ShuffleNet, MobileNetv2, DenseNet201	Ripeness level accuracy = 99.5% and Black Spot disease F-measure = 100%	[29]
Computer vision	Oranges, bananas, and apples	Rottenness	Normalization, data augmentation	-	CNN, MobileNetV2	Validation set accuracy = 99.61%	[30]
Computer vision	*Citrus*	*Citrus* disease defects	DCP, KF-2D-Renyi	Extract texture, edge, and shape features	ABC-SVM	Average recognition rate = 98.45%	[27]
Computer vision	*Citrus*	Disease	Normalization, image brightness adjustment, contrast enhancement		CNN	Accuracy = 89.1%	[103]
Computer vision	*Citrus*	Common *Citrus* diseases	Noise filtering, data augmentation, image segmentation	ECN, DOA	DOA-ECN-DSSAE	Accuracy = 98.4%	[136]
SIRI	Four types of *Citrus*	Rot	Image demodulation	-	CNN	Overall classification accuracy = 90.6%	[137]
HSI	Sugar Belle leaves and immature fruit	*Citrus* canker in various disease stages	-	-	RBF, KNN	RBF: asymptomatic accuracy = 94%; early accuracy = 96%; late accuracy = 100%	[44]
HSI	*Citrus*	*Citrus* Black Spot	SG, spectral calibration	PLS analysis	KNN	Healthy samples: accuracy = 100%; early disease samples: accuracy = 93.8%; late disease samples: accuracy = 80.2%	[138]
HSI	Oranges	Rot	Correction, ROI, threshold segmentation	PCA	PLS-DA, BP-ANN	Overall classification accuracy = 96.6%	[139]
MSI	*Citrus* fruit trees	Healthy and HLB-infected trees	Image stitching, liner stretch	PCA, autoencoder	SVM, KNN, LR, Naive Bayes, AdaBoost, Neural Network	AdaBoost: accuracy = 100%	[53]
MSI	Navel orange	Rot	BEMD	PCA	Improved watershed segmentation	Rotten fruits: accuracy = 97.3%; healthy fruits: accuracy = 100%	[140]
MSI	Newhall navel orange	Rot	Image correction	BOSS, BOSS-SPA, PCA	PLS-DA	BOSS-PLS-DA: accuracy = 97.1%; BOSS-SPA-PLS-DA: accuracy = 100%	[141]
Vis/NIR	Thompson and Jaffa oranges	Black rot, pH, TA, and SSC	SG, MN, SNV, CFS	PCA	SVM, BPNN	Thompson accuracy = 93%, Jaffa accuracy = 97%	[69]
NIR	*Citrus*	Hidden mold infection	De-bias, detrend, 1D, 2D, CWT, MM, MSC, SNV	PCA	PCA-FLD, SIMCA, SVM, PLS-DA	Detection accuracy = 100%	[67]
Raman	Orange and grapefruit leaves	Health, nutritional deficiencies, early and late HLB infection	Baseline correction, data normalization	-	OPLS-DA	Grapefruit: detection rate = 98%; orange tree: detection rate = 87%	[79]
Raman	Mandarin	Carotenoids and corruption	Polynomial smoothing and filtering, poly baseline correction	PCA	LDA, KNN, SVM	A. alternate: R_p_^2^ = 1.000; A. niger: R_p_^2^ = 0.900; P. italicum: R_p_^2^ = 0.800	[55]
Fluorescence imaging, MSI	Navel orange	HLB	Correction, ROI	-	MobileNetV3	Total accuracy = 96.5%	[142]
NIR, computer vision	*Citrus*	*Citrus* diseases	Data augmentation, normalization	-	Faster-CNN	Canker accuracy = 97%, Scab accuracy = 95%, Melanosis accuracy = 99%, HLB accuracy = 97%, Black Spot accuracy = 97%, healthy accuracy = 97%	[143]
NIR	Limone Costa d’Amalfi and Limone di Sorrento	Lemon equatorial diameter, peel thickness, juice yield, color; SSC, TA, pH, mineral content, and cation molar concentration	MSC, SNV, 1D, 2D	PCA, MLR, LDA	PCA, MLR, LDA	Distinguish between breeds and geographical origins	[144]
NIR	Different types of lemon juice	Adulteration	Mean centering, self-scaling processing	PCA	VIP-PLS-DA, CPANN	Accuracy = 96%	[145]
NMR	Sweet orange	62 ingredients in sweet orange	FT, phase adjustment, baseline correction	PCA	PLS-DA, OPLS-DA	Accurate classification of sweet oranges of different geographical origins	[92]
NMR	*Citrus* juice from San Pedro and Entre Ríos, Argentina	TA, carbohydrate, and signal from the ethanol region	-	PCA	PCA, PLS-DA	Accuracy = 100%	[93]
NMR	Orange and other four kinds of pure juice	Relative percentage of pure juice	Noise reduction, baseline correction, and normalization	Non-targeted approach	PLS	Orange: R^2^_P_ = 0.950, RMSEP = 4.435	[94]

1D: first derivative; 2D: second derivative; LR: Logistic Regression; CFS: correlation-based feature selection; SRC: Sparse Representation Classifier; GAN: Generative Adversarial Network; OTSU: OTSU Thresholding; SR: Square root Method; LM: Logarithm Method; IM: Inverse Method; RCA: Regression Coefficient Algorithm; MI-SPA: Mutual Information–Successive Projections Algorithm; MM: Min–Max Normalization; QDA: Quadratic Discriminant Analysis; SSOM: Supervised Self-Organizing Map; OPLS-DA: Discriminant Analysis of Orthogonal Partial Least Squares; MLR: Multiple Linear Regression; PSNV: Piecewise Standard Normal Variate; SPLINE: Spline Smoothing; CR: Continuum Removal; CAE: Convolutional Autoencoder; MSSS: Maximum Symmetric Surround Saliency Detection; CBAM: Convolutional Block Attention Module; FPN: Feature Pyramid Network; PAN: Path Aggregation Network; DCM: Diameter Correction Method; PSO-ELM: Particle Swarm Optimization–Extreme Learning Machine; MN: Mean Normalization; GLCM: grey-level co-occurrence matrix; DCP: Dark Channel Prior; ABC-SVM: artificial bee colony–support vector machine; BEMD: Bidimensional Empirical Mode Decomposition.

**Table 2:** Detection characteristics, advantages, and limitations of various computer vision and spectral technologies in *Citrus* fruit quality assessment.

Detection Technology	Spectral Range	Detection Characteristics	Advantages	Disadvantages
Computer vision	400–700 nm	External quality inspection. *Citrus* grading and classification. *Citrus* disease detection. Picking identification and positioning.	Simple operation, low-cost, fast, and wide application.	Data redundancy. Sensitive to external light. Image information depends on camera characteristics. Inability to detect internal quality.
HSI	200–2500 nm	Sugar content, acidity, hardness, maturity, flavonoids, and other natural active substances. Detection of agricultural product quality and defects. Disease detection.	Capable of simultaneously collecting images and spectral features to detect internal chemical composition information.	Data redundancy. High equipment cost.
MSI	400–1100 nm	Monitoring *Citrus* vegetation, water stress, and maturity.	Faster detection and lower equipment cost compared with HIS technology.	Low detection accuracy. Insufficient information for some specific tasks.
IR	780–1,000,000 nm	The most commonly used spectral technology for detecting the internal components of *Citrus*, such as SSC and TA, maturity, grading, and damage.	Simple operation, low-cost, fast, and can detect multiple chemical components in fruits with wide usage.	Large spectral range and requires chemometric knowledge to analyze.
Raman	0–4000 cm^−1^	Flavonoids. Disease detection.	Fast detection and high sensitivity.	Susceptible to interference from factors such as fluorescence, sample moisture content, and temperature. Limited detection range and high equipment cost.
Fluorescence spectroscopy	200–1000 nm	Fluorescent compounds. such as chlorophyll, flavonoids, carotenoids, acidity, and vitamin C.	Fast detection and high sensitivity.	Spectral analysis is complex. and the applicability of fluorescent groups is limited.
THz	0.1–10 THz	Flavonoid detection.	Low energy, strong penetration.	High equipment cost. Spectral features are difficult to distinguish.
NMR	1–900 MHz	Various ingredients of *Citrus*. Product traceability.	Fast, reproducible, and stable. High sensitivity.	Complicated operation and high sample processing.

## Data Availability

No new data were created or analyzed in this study. Data sharing is not applicable to this article.
